# The Economic Value of the Greater Montreal Blue Network (Quebec, Canada): A Contingent Choice Study Using Real Projects to Estimate Non-Market Aquatic Ecosystem Services Benefits

**DOI:** 10.1371/journal.pone.0158901

**Published:** 2016-08-11

**Authors:** Thomas G. Poder, Jérôme Dupras, Franck Fetue Ndefo, Jie He

**Affiliations:** 1 CRCHUS, CIUSSS de l’Estrie–CHUS, Sherbrooke, Québec, Canada; 2 GREDI, Université de Sherbrooke, Sherbrooke, Québec, Canada; 3 Département des sciences naturelles et Institut des sciences de la forêt tempérée, Université du Québec en Outaouais, Ripon, Québec, Canada; 4 Département d’Économique, Université de Sherbrooke, Sherbrooke, Québec, Canada; University of Waikato, NEW ZEALAND

## Abstract

This study used a contingent choice method to determine the economic value of improving various ecosystem services (ESs) of the Blue Network of Greater Montreal (Quebec, Canada). Three real projects were used and the evaluation focused on six ESs that are related to freshwater aquatic ecosystems: biodiversity, water quality, carbon sequestration, recreational activities, landscape aesthetics and education services. We also estimated the value associated with the superficies of restored sites. We calculated the monetary value that a household would be willing to pay for each additional qualitative or quantitative unit of different ESs, and these marginal values range from $0.11 to $15.39 per household per unit. Thus, under certain assumptions, we determined the monetary values that all Quebec households would allocate to improve each ES in Greater Montreal by one unit. The most valued ES was water quality ($13.5 million), followed by education services ($10.7 million), recreational activities ($8.9 million), landscape aesthetics ($4.1 million), biodiversity ($1.2 million), and carbon sequestration ($0.1 million). Our results ascribe monetary values to improved (or degraded) aquatic ecosystems in the Blue Network of Greater Montreal, but can also enhance economic analyses of various aquatic ecosystem restoration and management projects.

## Introduction

Ecosystems provide multiple benefits to human communities, from wetlands that act as buffer zones to help reduce flooding to woodlands that naturally filter the air by capturing dust. In addition to directly supplying basic goods and services such as food or firewood, ecosystems help regulate natural systems, are included as part of human culture and heritage and provide the basis for substantial contributions to the economy. Commonly referred to as ecosystem services (ESs), these elements are essential to human well-being and in many cases, cannot be replaced by manmade products [1–3].

The Millennium Ecosystem Assessment, a United Nations initiative that began in 2000, was designed to measure the effects of ecosystems changes on human well-being and is based on the concept of ESs [3]. The results of this initiative primarily show that improper valuation of natural capital has led to management decisions that have contributed to environmental degradation. Furthermore, this degradation threatens the future capacity of ecosystems not only to provide ESs but also to maintain the well-being of the beneficiaries of these services.

One explanation for the general trend of the deterioration of ESs is that a large proportion of these natural amenities are not connected to an existing economic market. Consequently, their price is null, which makes it difficult to include them in the economic system and in many cases has led to unsustainable usage of the ecosystems that provide them [4–5]. In economic terms, the negation of the scarcity or abundance of ESs, of their contribution to natural and human systems, and of their participation in societal welfare yields a fundamental imbalance in their usage [3–4]. Moreover, this imbalance misleads managerial planning and development of the territory in which these ecosystems function as this planning and development must confront important tradeoffs involving conservation, exploitation and the processing of natural environments [6]. This lack of consideration is reflected in the use of incentives and coping strategies that do not consider a realistic valuation of natural capital.

To measure the contribution of ESs to community well-being and to overcome the under-representation of natural amenities in the economic market, non-market economic valuation approaches have been developed [4,7]. This type of approach affixes ESs with a value that is quantifiable in economic or utility terms (i.e., a satisfaction measurement provided by the consumption of goods and services).

This context defines the issues and research questions of this study. Whereas an abundance of literature on the question of the economic value of ESs for terrestrial ecosystems has been produced, research on freshwater aquatic ESs remains limited [3–4,8]. It therefore seems appropriate to contribute to this research field by providing a valuation of ESs in the context of the Greater Montreal area (Quebec, Canada), a geographic space in which the river system is fundamental to the quality of life, the value of economic activities and the region’s cultural heritage.

In Canada, few studies have assessed the value of ESs provided by freshwater aquatic environments. These studies have shown that freshwater or marine systems contribute significantly both to the economy and to community well-being [9–10]. However, studies aiming to measure the total value of services provided within an ecosystem (i.e., multiple ESs representing direct, indirect and non-use values) are scarce.

This study aims to highlight the links between Greater Montreal’s freshwater aquatic ecosystems and human well-being by determining the economic value of the non-market ESs provided by these ecosystems. To accomplish this goal, we propose estimating these values by measuring variations in human welfare associated with three freshwater ecosystem-improvement projects in the Greater Montreal area using a contingent choice methodology.

This article is structured as follows: Section 2 presents the study site; Section 3 describes the contingent choice methodology and sampling strategy; Section 4 presents the results of willingness to pay for each ES; Section 5 discusses the results, stressing their interpretation and potential uses and underlining the limits of this study’s approach; and Section 6 concludes.

## Study Area

**Fig 1:**
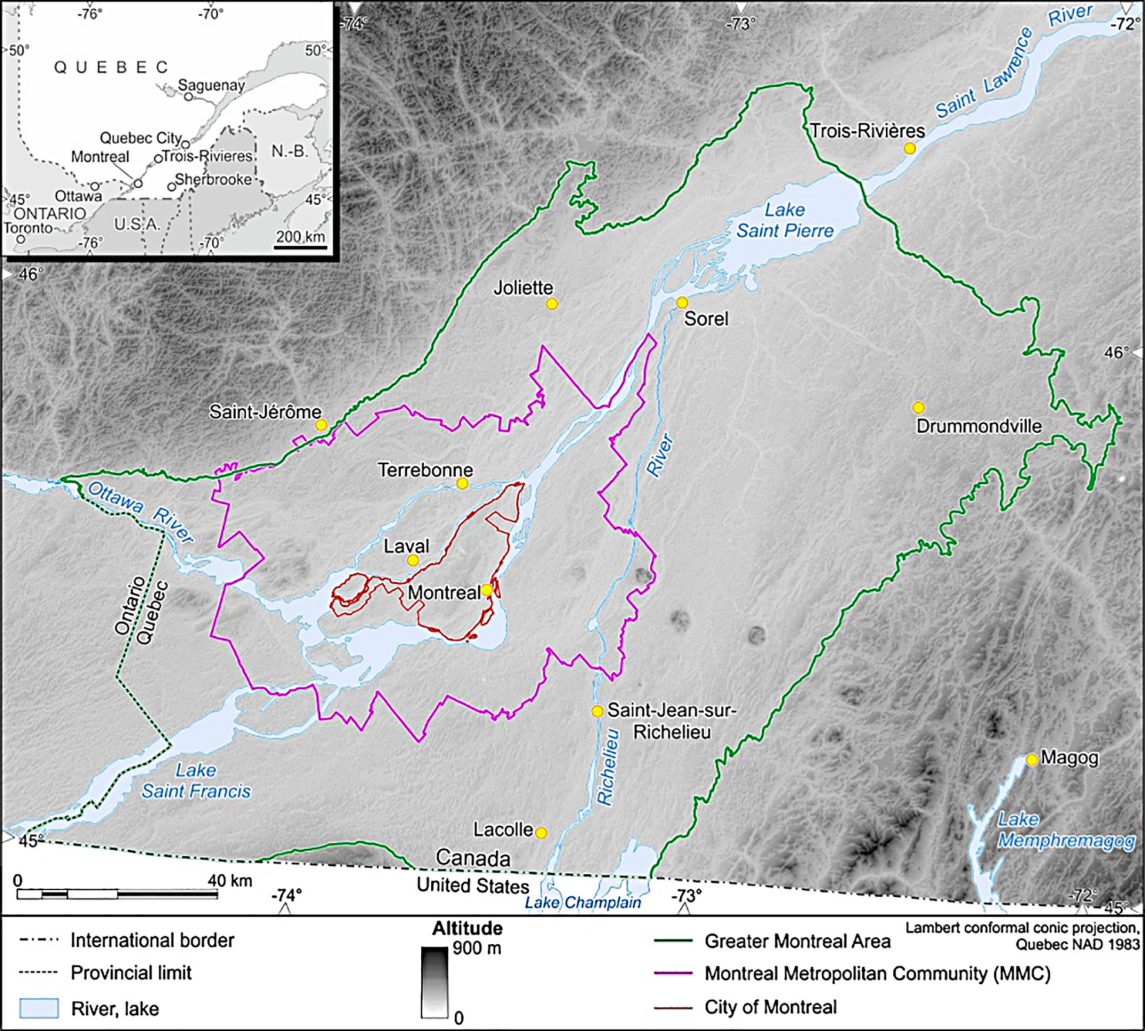
Location of the study site.

The territory covered by this study is the natural region of the Upper St. Lawrence Plain of Quebec’s ecological reference framework [11], often called the Greater Montreal Blue Network (GMBN). This area is not an administrative region, but an ecological zone with boundaries that are based on persistent elements of the natural landscape (i.e., geology, surficial deposits, relief, climate, drainage network, vegetation and wildlife) [11]. This area of more 1.7 million hectares overlaps two major bioclimatic regions–the maple-hickory and maple-basswood eco-regions–and includes the Montreal Metropolitan Community (a group of 82 municipalities including and surrounding the City of Montreal) and adjacent territories. The boundary of the area and its location in the province of Quebec are presented in Fig 1.

As described by Dupras et al. [12], the location and altitude of the study area endow it with a mild and humid climate conducive to a rich biodiversity. Although this area contains the greatest biodiversity in Quebec [13], it also endures the most human-generated stress on its natural systems. Urbanization, natural resource exploitation, agriculture, industrialization, environmental degradation and the introduction of invasive alien species are the major causes of the loss of biodiversity [14–15].

From a socio-economic perspective, this area covers only 1% of the territory of Quebec, but it is home to more than half of its human population (more than 3.9 million people) [16]. The myriad economic activities undertaken in the area include agriculture, leisure tourism, biotechnology, manufacturing, services, telecommunications, aerospace, information technology and pharmaceuticals. The City of Montreal is also a well-known scientific and cultural center [12,16].

**Fig 2:**
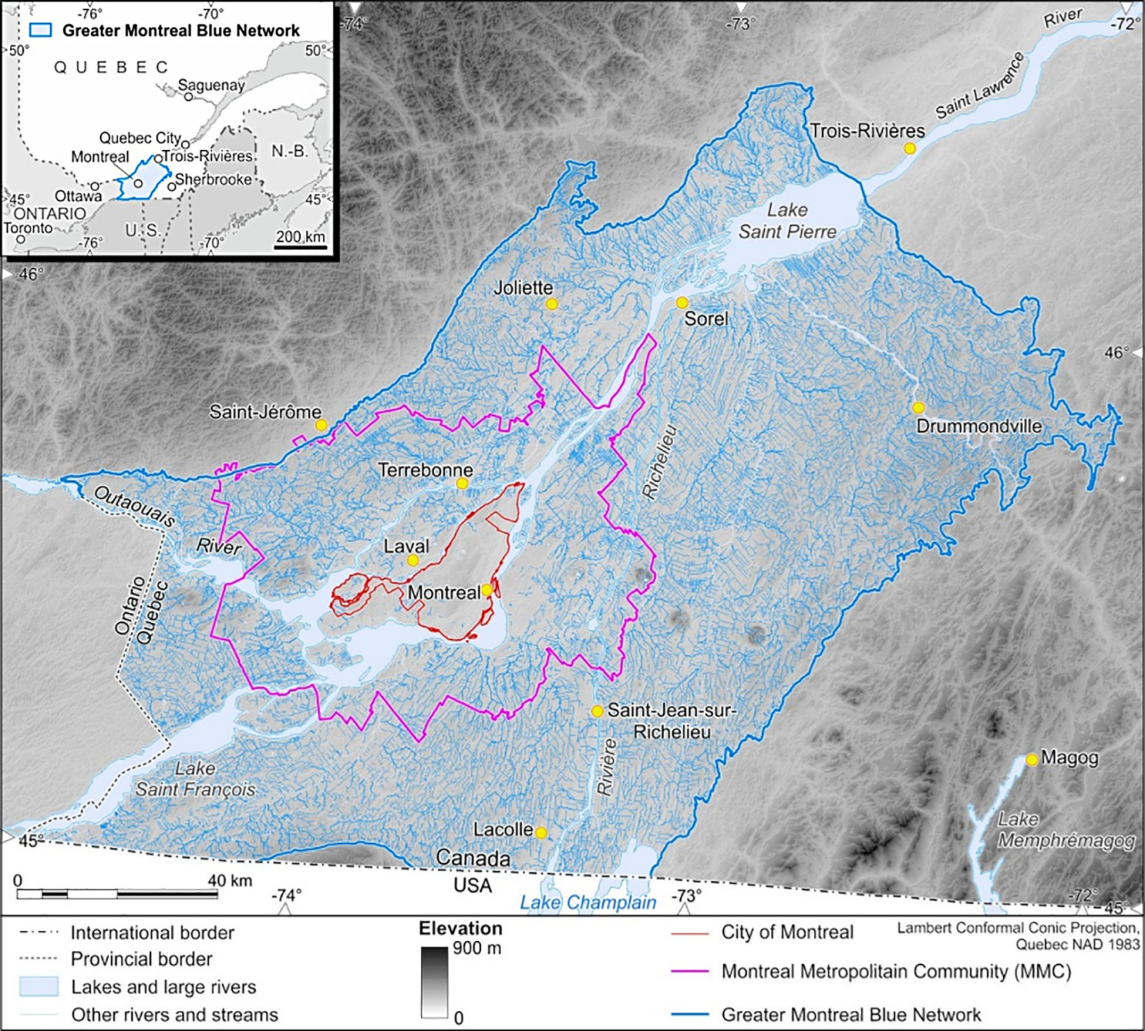
The bodies of water that constitute the Greater Montreal Blue Network.

Although it covers only 7.7% of the territory of the GMBN (132,561 hectares), the rich hydrographic system of the region (Fig 2) is one of its greatest assets in several respects: it catalyzes economic exchanges, contains high biodiversity and is an integral part of culture and heritage. The backbone of this network, the St. Lawrence River, spans an area in the province from the Ontario border to Lake Saint-Pierre and receives water from tributaries that originate in the Appalachian Mountains and the Canadian Shield.

## Materials and Methods

Many methods have been developed to provide an economic value to ESs that generally fall into five broad categories: methods based on market prices, methods based on costs, methods based on the revealed and stated preferences of economic agents, and methods based on benefit transfers [7].

In this study, we use a stated preferences method which asks people to choose their most favorite project among three real projects promoted by a non-profit organization called Nature Action Québec (NAQ). Our approach has similarity with the contingent ranking (CR) method and the choice experiment method (CE) [7,17,18]. Both methods are based on the random utility model (RUM) framework that was originally formulated by McFadden [19] and extended by Hanemann [20]. Similar to these approaches, our method is based on survey responses where individuals state their preferences for different scenarios presenting various improvements in current conditions with the help of specific attribute levels. However, different from the CR method, we do not ask respondents to rank a series of scenarios, and different from the CE method, the scenarios proposed are not hypothetical but real. Since our method asks people to make an hypothetical choice among real projects, we describe this one as a contingent choice (CC) method. The expressed tradeoffs between respondents’ assessments and the levels of the various attributes can then be used to estimate the implicit marginal value that respondents are willing to pay for each attribute. In this aspect, our method shares certain similarities with the choice experiment (CE) approach, except that the hypothetical scenarios presented in a CE are artificial combinations of attributes of different levels, whereas in our method, the scenarios typically involve extant environmental programs that are broken down into and presented as several components.

More precisely, in our study, a baseline scenario corresponding to the current situation is presented, along with three alternatives corresponding to three real projects at various stages of implementation in the study area. The three projects promoted by NAQ were carefully selected with the help of five field experts (four biologists and one land-use planner) to represent restoration projects that enhance the production of ESs in different ways. ESs were chosen after analyzing each restoration project and considering all ESs that might be either qualitatively or quantitatively measured. We convened focus groups with experts that enabled us to focus on six ESs: biodiversity, water quality, carbon sequestration, recreational activities, landscape aesthetics, and education. The levels of ESs before and after implementation of the restoration projects were assessed by means of a literature review and focus groups for each of the three projects. In addition to these ESs, two additional attributes were considered in the survey: the superficies of the area of restoration and the costs of the projects in Canadian dollars (CAD). The six points below describe the ESs that were analyzed and how their qualitative or quantitative levels were evaluated. Attributes and levels used in this study are listed in Table 1.

**Table 1:** Attributes and levels used in the contingent choice study.

Attributes	Description	Levels used in the study
Biodiversity	Number of plant and animal species	62, 163, 215*
Water quality	Classification for indicators of MDDELCC	Very bad, (bad), medium, good, very good
Carbon sequestration	Carbon sequestered (in tons)	1t, 30t, 59t
Recreational activities	Recreational opportunities provided by the improvement of the quality of the aquatic environment	2, 4, 5
Landscape aesthetics	Classification corresponding to the improvement of the quality of the landscape	(Low), medium, high
Education	Number of education activities	1, 2, 3
Area restored	Hectares	0.01ha, 1ha, 3.5ha
Cost	Suggested donation (in 2014 CAD)	0, 15, 20, 30, 35, 40, 45**

Notes: *The status quo was the baseline in each project and corresponded to the number of species before project implementation. In analyzing the data, only the variation between the status quo and the project's contribution was used

**Fig 3:**
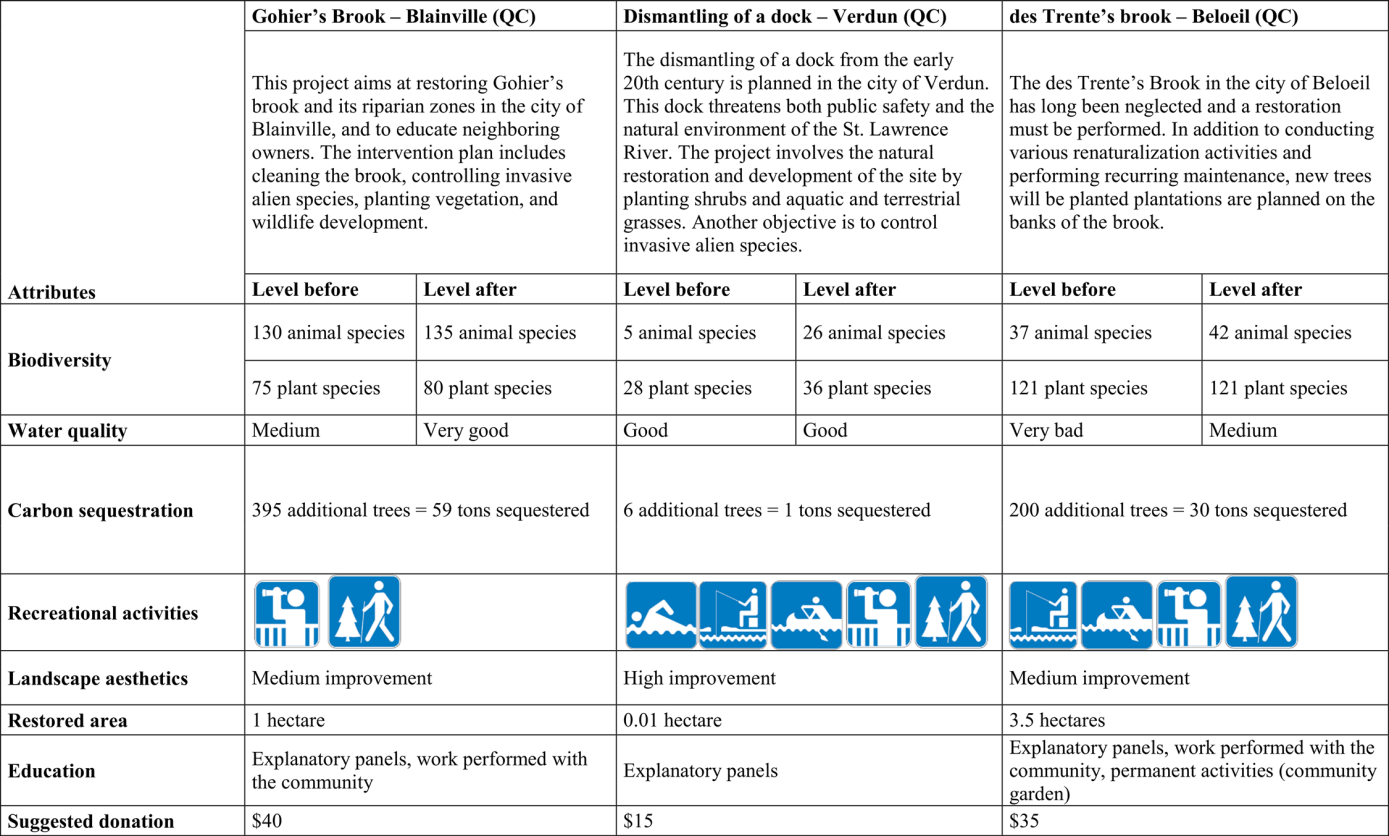
Scenarios presented to respondents in the contingent choice survey.

** The sample was randomly split in two, and the first subsample presented the costs in Fig 3 whereas the second subsample presented costs in terms of a donation of 20, 30 or 45 Canadian dollars. This was undertaken in an effort to create greater differentiation within the variable.

### i) Biodiversity

Biodiversity was measured by means of the number of plant and animal species. The goal, in terms of improved ES rendered, is to promote and secure the habitat of various animal and plant species and to control invasive species. The initial levels of species were assessed with field measurements. Post-restoration levels were determined by expert focus groups that considered the new habitats created.

### ii) Water quality

The measure of water quality improvement for different projects refers to the indicators of the Ministry of Environment of Quebec [11]. This classification scheme ranges from "very poor" to "excellent" and considers factors reflecting the quality of the aquatic environment, including the amount of fecal coliform per 100 milliliters, water temperature, pH, salinity, and dissolved oxygen levels. Improving water quality involves several interventions, such as planting riparian zones, stabilizing water banks and creating filtering marshes. Initial and final levels of water quality were assessed by expert focus groups and categorized under the Ministry of Environment classification.

### iii) Carbon sequestration

Improving carbon sequestration at the project sites primarily consists of planting new trees. Based on the previous literature [21–22] and expert opinion, we used a rate of 150 kg of carbon captured per tree in calculating the carbon sequestration capacity of various projects.

### iv) Recreational activities

This attribute refers to the recreational activities that will be enabled following implementation of a project, such as fishing, swimming, boating, pedestrian trails, and bird watching. An aquatic environment in better condition will permit a greater variety of recreational activities. The potential activities were listed by each project’s promoter and re-assessed by the focus groups.

### v) Landscape aesthetics

The quality of the landscape includes aesthetic criteria that shape its overall beauty. Based on interventions of various types (e.g., cleaning waterways, stabilizing shorelines) to improve the quality of the landscape as a whole and to promote the return to its natural appearance, the experts assessed qualitative levels of landscape aesthetics (from low to high amelioration).

### vi) Education

This attribute lists the activities that might be conducted after projects were implemented to raise public awareness of issues related to aquatic environments. As a component of these projects, these activities include involvement of the population in the projects (e.g., part of the restoration work is carried out by citizens), providing information and interpretation panels for the benefit of citizens, and planning permanent activities. Three levels were assigned to the projects based on the number of potential education activities that might be initiated.

For each project, there is an associated cost that is presented as a voluntary donation to an environmental NGO (i.e. non-governmental organization). Because the status quo involves no intervention, the associated cost was zero. For every other project, a donation amount was suggested based on the scale and the importance of the project with the goal of rendering the individual amount as reliable as possible. In most cases, the suggested donation was estimated using the actual costs of the projects. Fig 3 shows the details of the three projects that were presented to respondents. To reveal individuals’ WTP, respondents were asked to select their favorite project (including the status quo). The choice of one of the three restoration projects among the four alternatives proposed, expressed the respondents’ willingness to improve the ESs associated with the project in return for the cost of the project. In addition to the WTP questions, our questionnaire asked about respondents’ demographic and socio-economic characteristics, their knowledge regarding the contribution of aquatic systems to communities’ well-being, and their attitude toward environmental preservation.

The survey was conducted via Internet in 2014. The online survey firm SSI was retained to redirect respondents to the survey website through a random and representative sample of the target population (i.e., residents of Quebec over 18 years old). The sample is said to be representative since SSI used a quota sampling by age and sex. This survey and our study was approved by the ethics committee of the University of Sherbrooke and the CHUS.

Using U_ij_ to represent the related utility (or satisfaction) that can be obtained from project j (jϵJ, J = 1, 2, 3) by respondent i, we can write *U_ij_* = *βA_ij_* + *δC_ij_* + *γS_i_* + *ε_ij_*, where *A*_*ij*_ and *C*_*ij*_ are the vectors of the environmental attributes of and cost associated with a given project presented to respondent i, respectively, and *S*_*i*_ is a vector of individual characteristics. Project j will be chosen by respondent i if and only if U_ij_ >U_ik,_ kϵJ and k≠j. Regarding respondent i for project j among the J alternatives and in a conditional logit model, we can thus write the probability for a specific project j to be chosen by respondent i as follows:

$$prob(U_{ij}>U_{ik})=prob[\beta A_{ij}+\delta C_{ij}+\gamma S_{i}+\varepsilon_{ij}>\beta A_{ik}+\delta C_{ik}+\gamma S_{i}+\varepsilon_{ik})]=prob[(\varepsilon_{ij}-\varepsilon_{ik})>(\beta A_{ik}+\delta C_{ik})-(\beta A_{ij}+\delta C_{ij})]$$

By assuming that the error term (*ε_ij_*–*ε_ik_*) follows a Gumble distribution, the probability for an alternative j to be chosen among the J alternatives can be expressed in terms of a logistic distribution [19], also called a conditional logit model as follows:

$$prob(U_{ij}>U_{ik})=\frac{exp(\beta A_{ij}+\delta C_{ij})}{\sum_{k\epsilon J}exp(\beta A_{ik}+\delta C_{ik})}$$

The intuitive logic of the conditional logit estimation function is therefore to use the attributes of the project to explain the probability of a respondent choosing a project. Another detail that we observe in the above function is that individual i’s socioeconomic characteristics S_i_ are not directly identifiable in the function. By assuming that the socio-economic characteristics of the individual affects the coefficients β and δ, which measure the contribution of the attributes and the cost in the formation of respondent i’s utility (satisfaction) for a specific project, many authors employ the cross-term of project attributes *A*_*ij*_ and more recently, *C*_*ij*_, with the individual’s characteristics S_i_ [23]. We therefore have the following estimation function:

$$prob(U_{ij}>U_{ik})=\frac{exp[(\beta +{\alpha S}_{j})A_{ij}+\delta C_{ij}]}{\sum_{k\epsilon J}exp[(\beta +{\alpha S}_{j})A_{ik}+\delta C_{ik}]}$$

The marginal WTP of individual i for one unit of the improvement of a specific attribute t, tϵA can therefore be calculated as follows:

$${WTP}_{t,i}=-\frac{\hat{\beta_{t}}+\hat{\alpha_{t}}S_{j}}{\hat{\delta }}$$

where $\hat{\beta_{t}}$ and $\hat{\alpha_{t}}$ are the estimated coefficients for the terms related to the attribute t and $\hat{\delta }$ is the estimated coefficient for the attribute cost.

## Results

A total of 3,057 people were invited by SSI to complete the online survey, and 2,518 began the survey. This yielded a response rate of 82.4%. We excluded 369 persons who later determined that they were unwilling to be included in the database for personal reasons. A Thorough analysis of their characteristics whith those of the respondents included in the final analysis revealed no difference, except for the proportion of respondents living in the area of one of the three projects considered (i.e. 48% in the final sample versus 57%). Another 124 were excluded because they gave protest answers to explain their choice of the status quo in a follow-up question. We considered protest answers when respondents explained their reason for not choosing a project with the following reasons: “Money should be better spent as a priority, such as for health care” (n = 77), “I should not have to pay for a problem created by others” (n = 34), “I do not trust the non-governmental organization to appropriately manage the funds” (n = 29), and “The projects do not make sense to me” (n = 12). In addition, 283 questionnaires were unusable because they were incomplete. Consequently, data from 1,742 respondents were included in our analysis (i.e. 69% of data collected). Considering that we used a conditional logit for a given choice among four alternatives, this yielded 6,968 points of observation in the estimates. The data were collected in March 2014. Descriptive statistics for the 1,742 respondents are given in Table 2.

**Table 2:** Descriptive statistics of variables used in the analysis.

Variable	Description	Mean (SD)
Biodiversity	Scenario attribute: Additional plant and animal species	11 (10.98)
Water	Scenario attribute: Improvement in water quality^a^	0.67 (0.82)
Carbon	Scenario attribute: Carbon sequestered in tons	22.5 (24.28)
Recreational	Scenario attribute: Number of recreational opportunities	2.75 (1.92)
Landscape	Scenario attribute: Improvement in landscape quality^a^	1 (0.71)
Education	Scenario attribute: Number of education activities	1.5 (1.12)
Area	Scenario attribute: Area restored in hectares	1.13 (1.43)
Cost	Donation proposed for each project (Canadian dollars)	23.13 (16.19)
Age	Age (in years)	46.42 (15.26)
Sex	Female = 0, Male = 1	0.48 (0.50)
Income	Household income before taxes (Canadian dollars)	53,706 (36,188)
Left	Voted for a left-wing political party in the most recent election	0.43 (0.50)
Urban	Whether respondent has lived most of his/her life in an urban area (yes = 1, no = 0)	0.70 (0.46)
Aquatic	Whether respondent regularly practices one of the three aquatic activities (i.e., swimming, fishing, boating) (yes = 1, no = 0)	0.59 (0.49)
Bad_AE	Whether respondent believes the aquatic ecosystem is very deteriorated (yes = 1, no = 0)	0.87 (0.34)
Indiv_resp	Whether respondent believes that individuals can contribute to a better environment (yes = 1, no = 0)	0.64 (0.48)
Donate	Whether respondent has previously donated to an environmental fund (yes = 1, no = 0)	0.29 (0.45)

Note: SD means standard deviation.

^a^ These improvements indicate a change in quality level. On average in each scenario proposed, the water quality increase by 0.67 level (e.g. from medium to almost good) and the landscape quality by 1 level (e.g. from medium to high). See table 1 for the levels used.

Table 3 presents the results of our estimates with a conditional logit. The first estimate presents a full specification with attributes and interactive terms with covariates. The second estimate drops interactive terms that are not significant at the 10% level. The coefficients obtained in this second estimate are used to calculate the marginal WTP for each attribute (i.e., for each ES). In these estimates, not all the attributes were considered due to perfect multicollinearity between certain attributes. Indeed, we have been able to include the attributes of biodiversity, water quality and carbon sequestration in our estimates. Because we chose to utilize real projects in this study, we have no control over this multicollinearity. However, we can still derive the marginal value for each attribute excluded from the estimation of choice function by using the perfect linear correlation between the attributes excluded from the estimation function and those included. This also means that we can only include the attribute that contributed to the choice decision function in the calculation of the total WTP for each scenario.

**Table 3:** Conditional logit estimates.

Independent variables	Model 1		Model 2	
	Coefficient	P-value	Coefficient	P-value
Biodiversity	0.0012	0.929	0.0051	0.705
Biodiversity*Age	-0.0011	0.000***	-0.0011	0.000**
Biodiversity*Sex	-0.0118	0.013**	-0.0099	0.025**
Biodiversity*Income	0.0000	0.040**	0.0000	0.160
Biodiversity*Left	0.0083	0.084*	0.0072	0.113
Biodiversity*Urban	0.0088	0.094*	0.0073	0.145
Biodiversity*Aquatic	0.0113	0.019**	0.0113	0.014**
Biodiversity*Bad_AE	0.0286	0.000***	0.0287	0.000***
Biodiversity*Indiv_resp	0.0112	0.022**	0.0106	0.019**
Biodiversity*Donate	0.0321	0.000***	0.0320	0.000***
Water	0.1193	0.545	0.2324	0.151
Water*Age	-0.0093	0.000***	-0.0094	0.000***
Water*Sex	-0.0654	0.378		
Water*Income	0.0000	0.336		
Water*Left	0.0512	0.489		
Water*Urban	0.0721	0.363		
Water*Aquatic	-0.0072	0.925		
Water*Bad_AE	0.3056	0.008***	0.3143	0.001***
Water*Indiv_resp	0.0471	0.533		
Water*Donate	0.2229	0.007***	0.2218	0.006***
Carbon	0.0065	0.467	0.0067	0.401
Carbon*Age	-0.0002	0.004***	-0.0003	0.002***
Carbon*Sex	0.0001	0.965		
Carbon*Income	0.0000	0.268		
Carbon*Left	0.0073	0.003***	0.0081	0.000***
Carbon*Urban	-0.0057	0.032**	-0.0044	0.058*
Carbon*Aquatic	0.0058	0.028**	0.0056	0.014**
Carbon*Bad_AE	-0.0003	0.929		
Carbon*Indiv_resp	-0.0014	0.597		
Carbon*Donate	0.0070	0.011**	0.0070	0.009***
Cost	-0.0096	0.414	-0.0094	0.423
Number of observations	6968		6968	
LR chi2	237.72		231.27	
Prob > chi2	0.0000		0.0000	
Pseudo R2	0.0492		0.0479	

Note: *** indicates a significant result at p<0.01

** at p<0.05

* at p<0.1.

The results in Table 3 indicate that the estimated coefficients for the included attributes have the expected signs. The higher the number of species proposed to be saved, the higher the level of targeted water quality improvement and the larger the promised carbon sequestration, the higher the likelihood that a project will be chosen. As expected, a more expensive project will have a lower likelihood of being chosen. The low statistical significance found for the attribute of cost can be explained by the relatively small differentiation in the amounts of required donations for different projects, which vary from 15 to 45 dollars.

The interactive terms between the projects’ non-monetary attributes and the respondents’ socio-economic characteristics reveal how a person’s awareness of an attribute can be affected by his/her socio-economic characteristics. Our results show that younger female respondents with higher incomes who have previously voted for left-wing political parties, who have lived most of their lives in urban areas, who frequently practice aquatic recreational activities, who have previously noticed the deterioration of the aquatic environment in Quebec, who believe that protecting the environment is the responsibility of each individual and who have a history of donating to environmental causes are more sensitive to the proposed improvement in biodiversity. Given a consistent level of proposed water quality improvement, younger respondents who frequently practice aquatic recreational activities, who have noticed the deterioration of aquatic environment and who have previously donated to environmental causes will have a greater probability of supporting one of the proposed projects. Finally, with respect to carbon sequestration, younger respondents who have previously voted for a left-wing party, who have lived most of their life in urban areas, who frequently engage in aquatic recreational activities and who have previously made environmental donations are more likely to support one of the proposed projects.

Estimated changes in the aquatic ecosystems caused by implementation of the various projects allowed us to quantify the marginal value of each of the selected attributes. In so doing, we value the improvement of the GMBN instead of attributing an overall value to it. The marginal value that we calculated represents the WTP, by means of a donation, that each respondent in a household was willing to pay to increase the ES of one unit. Thus, the marginal value of biodiversity (Table 4) can be defined as the maximum amount that a respondent is willing to pay to increase the level of biodiversity of one species (i.e., $1.36). Similarly, a respondent was willing to pay $0.11 for each ton of carbon sequestered and $24.53 for each additional hectare restored. With respect to water quality, a respondent was willing to pay $15.39 for improving water quality to the next level (e.g., "bad" if the initial quality was "very bad"). For landscape aesthetics and recreational activities, these marginal values were $4.69 and $10.16, respectively. Given the economic values estimated for each attribute’s unit, it was possible to infer a value that each household was willing to pay to finance the environmental benefits associated with the proposed projects (Table 5).

**Table 4:** Marginal willingness to pay per household aggregated across Quebec citizens.

Attributes	Marginal WTP/household ($)	WTP across Quebec (k$)
Biodiversity	1.36/species	1,187/species
Water quality	15.39/level	13,466/level
Carbon sequestration	0.11/ton	0,096/ton
Recreational activities	10.16/activity	8,890/activity
Landscape aesthetics	4.69/level	4,104/level
Restored area	24.53/ha	21,464/ha
Education	12.27/activity	10,736/activity

Note: values are expressed in 2014 Canadian Dollars

**Table 5:** Total non-market economic value per household for each restoration project.

	Blainville		Verdun		Beloeil	
Attributes	Change in levels	Total value ($/hh)	Change in levels	Total value ($/hh)	Change in levels	Total value ($/hh)
Biodiversity	10 species	13.56	29 species	39.32	5 species	6.78
Water quality	2 levels	30.78	0 level	0	2 levels	30.78
Carbon sequestration	59 tons	6.49	1 tons	0.11	30 tons	3.30
Recreational activities	2 activities	20.32	5 activities	50.80	4 activities	40.64
Landscape aesthetics	2 levels	9.38	3 levels	14.07	2 levels	9.38
Restored area	1 ha	24.53	0.01 ha	0.25	3.5 ha	85.86
Education	2 activities	24.54	1 activities	12.27	3 activities	36.81

Note: hh is for household.

The values in Table 4 for residents of Quebec were obtained by applying the marginal WTP per household by the 3.5 million households in Quebec [24]. We also applied a weighting of 0.25, which corresponded to the proportion of respondents who reported being ready to make a real donation to fund their favorite project. This weighting allowed us to better estimate our respondents actual WTP to improve the GMBM’s ecological health.

## Discussion

The values we found converge with other WTP estimates related to aquatic ESs in Canada and elsewhere. For example, using a CE method to value ESs provided by wetlands in Quebec, He et al. [25] found an annual household marginal WTP of $1.07 for one endangered species in less (i.e. a reduction in the number by one) and $1.24 for one CFU reduction in fecal coliform per 100 mL (from 100 to 0, yielding $124). In our survey, such an improvement in water quality corresponds to a change from a very bad level to a very good level, i.e., $61.56. In studying the WTP for environmental services for a lake in Florida, Shrestha et al. [26] found annual values of US $30.24 and US $71.17 per household for a moderate and a high improvement in water quality, respectively. The moderate improvement corresponded to a reduction in phosphorus runoff anywhere between 31% and 60%, whereas the high improvement was for a reduction between 61% and 90%. These values are similar to those found in our study. Shrestha et al. [26] evaluated their other attributes differently than we do, which makes a comparison difficult (i.e., improvement from limited to high for wildlife habitat and carbon sequestration), unless one considers that the increase in the number of species in the Verdun project is a high improvement in our study–in such a case, we have a WTP of $39.32, which is close to the WTP of Shrestha et al. [26] (i.e., US $41.06). Farber and Griner [27] found a WTP range from US $75.63 to US $112.44 per household per year, using a conjoint analysis to value stream improvement (mainly water quality) from severely polluted to unpolluted in western Pennsylvania.

In Europe, using a CE to value ESs from different types of bodies of water in Ireland, Doherty et al. [28] found marginal WTP that ranged from €15 to €25 per person per year for improvements in the health of ecosystems (i.e., biodiversity). For water clarity and smell, these values ranged from €30 to €46, whereas they ranged between €11 and €14 for access to recreational activities. These values were calculated for improvements from a poor level to a moderate or a good level and were slightly higher than those obtained in our study. Brouwer et al. [29] have found a WTP ranging from €157.80 to €225.40 per household per year for an improvement in the water quality of a river basin in Spain from current to very good. However, these values included not only water quality but also recreational and agricultural activities. Finally, using a CR method to value water quality improvements (small, medium and large improvement) in an urban river in the United Kingdom, Bateman et al. [17] have found a WTP ranging from £8.64 to £31.5. However, that study describes water quality as a mix of fishing, plants and wildlife, and boating and swimming, which may nonetheless correspond to the sum of our study’s categories of water quality, biodiversity and recreational activities.

Moreover, the respondents’ ranking of ES preferences is consistent with recently performed ES valuation studies in Quebec. For example, He et al. [25] revealed that water quality collected the highest WTP, followed by biodiversity, flood control and carbon storage. This ranking was also highlighted in a study by Dupras et al. [30], which used the CE method; in that study, respondents expressed a significant WTP for water quality improvement, followed by landscape aesthetics and fish biodiversity enhancement in the context of a watershed restoration by implementing agri-environmental practices. Notably, this study was conducted in a watershed located inside the GMBN. Furthermore, in reviewing the demand for agriculture and countryside attributes, Hall et al. [31] generally found that the highest demand for non-market ES is related to water quality improvement, followed by patrimonial considerations, landscape aesthetics and the protection of wildlife habitat.

Considering the growing demand by practitioners for non-market ES values, we believe that the values found in this study are robust and can be a useful source for public and private decision-makers who are utilizing benefit-transfer approaches. This institutional demand is, for example, illustrated by ecosystem accounts that have been initiated by both the Canadian and the Quebec governments [32–33]. These approaches to ecosystem accounting and valuation seek to establish several measures of the quantity and quality of both ecosystems and the services they generate; notably, they highlight the importance of producing more non-market economic values for ESs.

Nevertheless, our approach is somewhat limited. Its first limitation relates to the number of ESs analyzed. This limitation is related to both the nature of the method and the scale of our analysis. First, our CC method refers to respondents’ evaluation of the hypothetical future of real projects compared with the status quo alternative. In our study, the future design of the restored sites was assessed by expert focus groups to the best of their knowledge. However, it was impossible to make precise statements about several ESs; to do so would have required both precise field measurements to establish the starting point and exhaustive modeling to predict a fairly good level of ES after implementation of the projects. This explains why we could not measure certain important supporting and regulating services commonly related to freshwater systems, such as nutrient filtration or water yield. Second, the scale used for the projects in the valuation model is also limiting. For some ES, it is difficult to consider a small area (for example, the 0.01 to 3.5 hectares that we considered in our study) that is separate from the rest of the environment. For example, water quality can be improved by the three projects, but these improvements will be rapidly diluted in the rest of the water system, which creates a particular conflict with the aggregation of values to other scales or the transfer of values to other sites.

The advantage of the valuation method we used, in addition to being able to value most of the ES related to GMBN, is that it provides an indication of the relative importance that people attach to different attributes. However, while ranking the different attributes based on the reported WTP in our study, it must be remembered that the levels for each attribute do not have identical importance. Indeed, a higher level of water quality is a substantial change whereas a higher level of biodiversity (an additional species) might be thought to represent a marginal change, particularly if the baseline situation (the status quo) already contains several dozen observable species.

An advantage of the valuation method chosen is also the ability, by means of the WTP unit, to attribute a monetary value to each project’s non-market, environmental benefits (Table 5). In this setting, the improvements that an ecosystem environmental project will achieve are straightforward. This type of information is useful for decision-makers and planners working within budget constraints to maximize the benefits of publicly or privately financed projects. However, considering that we could not value more than six ESs, it must be emphasized that our results may constitute a lower bound of the non-market values associated with aquatic ecosystems. Consequently, readers who use these results should bear in mind that unassessed ESs also provide benefits and should be considered together with other indicators (e.g., social, biophysical) in the decision-making processes. Moreover, more exhaustive work is required to provide additional economic indicators that achieve a more comprehensive reflection of natural systems’ overall economic value.

## Conclusion

In addition to important provisioning services such as fish production and freshwater supply, the GMBN’s freshwater ecosystems produce a large spectrum of non-market ESs. Whereas some of these services are easily quantifiable, others–because of the intangibility of their benefits–are not. This situation is likely to endanger not only these ecosystems’ sustainability but also communities’ capabilities to meet their basic needs. Initiatives at various levels have therefore emerged both to compensate for this lack of representativeness and to promote the consideration of non-market services that are provided by ecosystems in decision making. To this end, ES valuation studies have primarily been developed to assess terrestrial ecosystems, thus leaving aquatic ecosystems uncharted. It is in this context that this study was proposed.

To provide economic indicators that reflect non-market benefits, we measured the economic value of six different non-market ESs. These ESs range from regulating services (carbon sequestration and water quality) to habitat services (biodiversity) to cultural services (education, landscape aesthetics and recreational activities). Under certain assumptions, the study’s results established that in the context of the restoration of three sites in the GMBN, Quebecers would be willing to pay $1.2 million to achieve a one-level improvement in biodiversity (one species), approximately $13.5 million to improve water quality and a little under $0.1 million to improve carbon sequestration capacity. Moreover, they would be willing to pay slightly less than $8.9 million to benefit from new recreational activities, more than $4.1 million to improve the quality of landscape aesthetics and approximately $21.5 million for larger restored areas. Finally, they would be willing to give more than $10.7 million dollars for environmental promotion activities to integrate and sensitize the local population.

Although it does not provide an overall value of GMBN, the WTP of households obtained in this study are important and provide a monetary value for any improvement or deterioration in the quality of the GMBN’s ecosystems. Indeed, many projects designed to improve the quality of aquatic system-related ecosystems either have been or are being implemented. The results of this study thus not only offer the possibility to quantify the nonmarket benefits of projects related to aquatic ecosystems in monetary terms but also provide a new perspective on environmental planning and management projects in the GMBN.
